# Prognosis, Immune Microenvironment Infiltration and Immunotherapy Response in Clear Cell Renal Cell Carcinoma Based on Cuproptosis-related Immune Checkpoint Gene Signature

**DOI:** 10.7150/jca.88467

**Published:** 2023-10-09

**Authors:** Gang Liu, Feifei Li, Yuntian Ge, Yaxing Shi, Fang Ren, Liancheng Zhu

**Affiliations:** 1 Department of Urology, Shengjing Hospital of China Medical University, Shenyang 110004, Liaoning, China.; 2 Department of Gynecology, Shandong Provincial Hospital Affiliated to Shandong First Medical University, Jinan, China.; 3 Department of Obstetrics and Gynecology, Shengjing Hospital of China Medical University, Shenyang, Liaoning, China.

**Keywords:** immune checkpoint, cuproptosis, clear cell renal cell carcinoma, immune microenvironment, prognosis, immune therapy.

## Abstract

**Background:** Immune checkpoint genes (ICGs), which are the cornerstone of immunotherapy, influence the incidence and progression of clear cell renal cell carcinoma (ccRCC). It is important to note that there is not much data in the literature to determine how cuproptosis and antitumor immunity are related.

**Methods:** On the basis of The Cancer Genome Atlas ccRCC dataset (n=526), cuproptosis-related ICGs (CICGs) were used to identify distinct molecular subtypes. Using the Cox regression method, a risk signature was constructed and externally validated using the ICGC (n=91) and primary ccRCC subgroups of GSE22541 (n=24). The molecular and immune characteristics and efficacy of immunotherapy in the subgroups defined by the risk score were investigated. Four risk CICGs were verified through in vitro experiment.

**Results:** We identified two unique molecular subgroups with substantial prognostic differences based on 17 CICGs. The two subtypes clearly differ in terms of the tumor immune microenvironment (TME). A predictive risk signature (CD276, HLA-E, LGALS9, and TNFRSF18) was created and externally confirmed, and their expressions were validated by realtime PCR. The multivariate Cox regression analysis demonstrated that this signature could independently predict survival. Thus, a credible nomogram incorporating the signature, age, stage, and grade was constructed, and discrimination was confirmed using the C-index, calibration curve, and decision curve analyses. The underlying implications for immune checkpoint inhibitors, the landscape of the TME, and single-cell level localization are depicted. In addition, its accuracy in forecasting actual immunotherapeutic results has been proven (CheckMate025 and TCGA-SKCM cohorts). The sensitivity of the two risk groups to various drug-targeted therapy methods was analyzed.

**Conclusions:** The data provided here provide the groundwork for creating customized therapeutic options for individuals with ccRCC. The findings also suggested that researching the cuproptosis-based pathway might improve ccRCC patient better prognosis, development of anti-tumor immunity, and therapeutic strategies for immunotherapy.

## Introduction

Renal cell carcinoma (RCC) is the fourteenth most prevalent disease in women and the ninth most common cancer in men in the world, and its increasing morbidity and mortality have raised serious concerns 1. Despite the rapid advancements and extensive clinical utilization of antiangiogenic medications and immune checkpoint inhibitors (ICIs) in the management of cancer, including RCC, the median overall survival (OS) rate of RCC patients remains suboptimal 2. Clear cell renal cell carcinoma (ccRCC) is the predominant histological subtype of RCC, constituting approximately 75-80% of all diagnosed cases of RCC 1. The use of ICI in immunotherapy has had a transformative impact on the treatment of ccRCC 3. In 2021, the National Comprehensive Cancer Network (NCCN) issued a favorable rating and recommended ICI-based therapy as a first-line treatment for metastatic RCC 4. Additionally, a well-conducted clinical trial has reported encouraging outcomes with ICI therapy, specifically anti-PD1 agent Pembrolizumab, in the perioperative (adjuvant or neoadjuvant) settings for ccRCC 5. Due to the intricate etiology, distinctive characteristics of the tumor immune milieu in ccRCC, and the presence of tumor heterogeneity, a considerable number of patients exhibit resistance to ICI therapy. Hence, the ability to forecast the individual response of patients to immunotherapy can be achieved through the analysis of molecular or gene signatures and the utilization of specialized models. The association between immune checkpoint genes (ICGs) and the progression and metastasis of cancer has been established, suggesting that ICGs could serve as potential targets for ICI therapy. The examination of existing clinical and expression data pertaining to the combination of ICGs can contribute to the discovery of personalized therapeutic targets and the enhancement of current treatment strategies.

Copper is a significant nutritional component that possesses oxidation-reduction (redox) properties, facilitating the development and proliferation of cells dependent on copper (cuproplasia), as well as inducing mitochondrial-mediated cell death (cuproptosis) beyond a specific threshold 6. Copper-induced cell death refers to the mechanism by which copper molecules directly interact with lipoylated constituents of the tricarboxylic acid cycle. This interaction leads to the aggregation of lipoylated proteins and subsequent impairment of iron-sulfur cluster proteins. Consequently, proteotoxic stress ensues, ultimately resulting in cellular demise. This phenomenon has been demonstrated by Tsvetkov et al. in their research 7. The researchers discovered that the process of cell death triggered by copper is distinguishable from all established regulatory mechanisms of cell death, such as apoptosis, ferroptosis, pyroptosis, and necroptosis. The authors proposed the term "cuproptosis" 7 to designate this previously unidentified mechanism of cell death. The relationship between cuproptosis and antitumor immunity remains unexplored in current scientific literature. The investigation of the co-expression association between genes associated to cuproptosis (CRGs) and immune checkpoint genes (ICGs) can provide valuable insights into the interplay between cuproptosis and the immune response against tumors.

**Figure 1:**
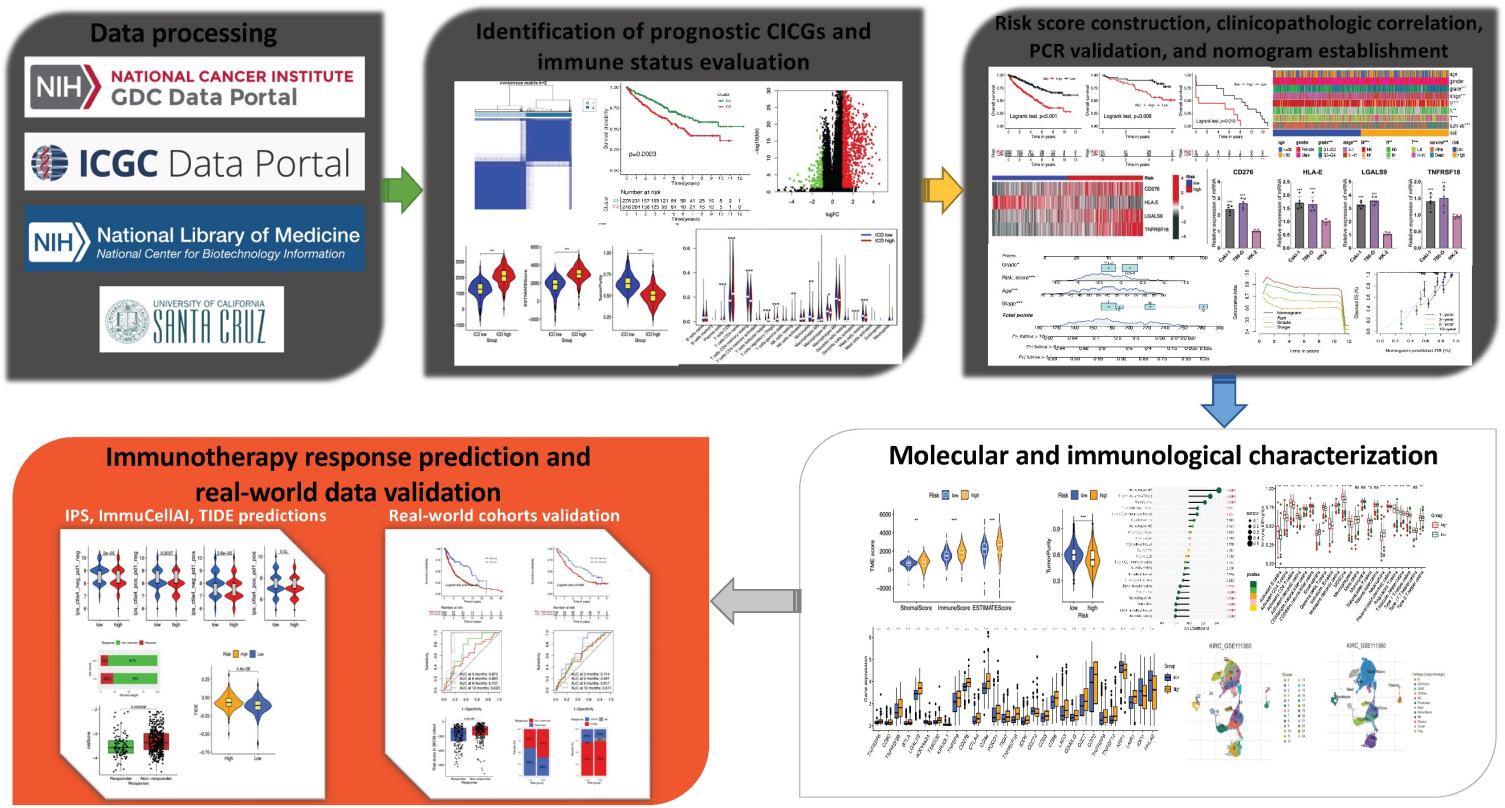
Workflow of the study.

The present study examined the expression levels of cuproptosis-related ICGs, investigated the association between ICGs and the prognosis of ccRCC patients, explored the influence of the tumor microenvironment (TME), and assessed the responsiveness to immunotherapy. The aforementioned results have the potential to make significant contributions towards the advancement of tailored therapy for ccRCC. The methodology employed in this investigation is visually depicted in Figure 1.

## Materials and Methods

### Data source

The Cancer Genome Atlas (TCGA) (https://portal.gdc.cancer.gov) was utilized to collect RNA-seq (FPKM) data for ccRCC (n = 539) and neighboring nontumorous kidney (n = 72) samples. The clinicopathological characteristics of patients with ccRCC (n = 526), including OS, were also obtained from TCGA database. Based on the patient identification number, we checked the patients' transcriptome data with their clinical information and excluded the data for patients whose information did not match. Complete gene expression profiles were obtained for 526 patients with ccRCC. We accessed the International Cancer Genome Consortium (ICGC) database (https://dcc.icgc.org/projects/LIRI-JP) to obtain the mRNA expression profiles (normalized read count) and clinical information of ccRCC patients (n=91). GSE22541 contains 68 patients, of whom 24 had a primary ccRCC diagnosis. This study employed data from 24 patients in the GSE22541 dataset to verify a risk model. We used GSE73731, which compares 265 ccRCC patient datasets, to validate the risk model. Pancancer data downloaded from the Xena database (https://xenabrowser.net/) were used for risk model prognosis validation. We received the raw "CELL" file from GEO (https://www.ncbi.nlm.nih.gov/geo/), normalized the quantiles, and adjusted the background. The batch impact of the merged dataset is then removed using SVA's R tool.

### Identification of cuproptosis-related immune checkpoint genes

In previous research, thirteen cuproptosis-related genes (CRGs) 7 and 79 immunological checkpoint genes (ICGs) 8 were identified; the entire list of these genes is included in Table S1. The association between the expression of CRGs and related ICGs in malignant tissues of the TCGA ccRCC cohort was then determined by calculating Pearson's correlation coefficients. Cuproptosis-related immune-checkpoint genes (CICGs) were discovered using p<0.0001 and Pearson's correlation coefficient absolute values larger than 0.30.

### Unsupervised clustering for differentially expressed prognostic CICGs

Using the "edgeR" R package, precancerous and cancerous tissues in TCGA cohort were screened for differentially expressed CICGs. The screening criteria were false discovery rate (FDR) <0.0001 and |log2FC|≥1. Differential expression was visualized using a heat map. Among the CICGs, predictive genes were identified using univariate Cox regression analysis (p <0.05). Based on these predictive CICGs, a consensus unsupervised clustering analysis approach was applied to identify distinct molecular groupings. To cluster the data, the "ConsensusClusterPlus" R tool was used, and the following criteria were applied: first, the curve of the cumulative distribution function (CDF) expanded gradually and smoothly; second, there were no groups with small sample numbers. Clustering ultimately increased the intragroup correlation while lowering the intergroup correlation. Using a heatmap, the differential expression patterns of CICGs among clusters were revealed. Using the Kaplan-Meier curve created by the "survival" and "survminer" R packages, the variance in OS between subtypes was analyzed.

### Differentially expressed genes identification and functional annotation

Using R's "limma" package with a fold-change threshold of 2 and an adjusted p-value of 0.05, the differentially expressed genes (DEGs) between clusters were discovered. The DEGs were shown using a volcanic plot. Using the "clusterprofiler" package in R, functional enrichment analysis was performed on DEGs to further analyze their likely activities and identify associated gene functions and enriched pathways.

### Construction of the cuproptosis-related immune-checkpoint gene signature

To avoid overfitting the model, univariate variables (p < 0.05) were included in the least absolute shrinkage and selection operator (LASSO) analysis, which was used to further select important predictive features. The optimized model was developed using a 10-fold cross-validation strategy. After calculating the risk score, the following regression coefficients were calculated:

Risk score = 
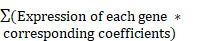


Based on the median risk score, the patients in TCGA cohort were separated into low- and high-risk groups, and OS was compared between the groups using the Kaplan-Meier method and log-rank test. Using the "survivalROC" R package, the area under the time-dependent ROC curve (AUC) was calculated to measure the prediction accuracy of the risk score. To validate this prognostic model, the risk score for each patient in the external test cohorts (ICGC and primary ccRCC subgroup of GSE22541) was generated in the same way to ensure the consistency of the model.

### Cell Culture and Treatment

The human renal proximal tubule epithelial cells (HK-2 cells) and renal clear cell carcinoma cell lines (786-0 and Caki-1) were procured from the American Type Culture Collection (ATCC) located in Manassas, VA, United States. The cells were subjected to incubation at a temperature of 37°C in an environment with 5% CO2 concentration and high humidity. They were regularly grown in RPMI 1640 or DEM, both of which were supplemented with 10% fetal bovine serum (Invitrogen, Carlsbad, CA, USA).

### RNA Extraction, Reverse Transcription, and Quantitative Real-time PCR (qRT-PCR)

A spectrophotometer is used to determine the quantity and quality of total RNA after it has been extracted from the aforementioned cells using the total RNA extraction micro-Kit (RNT411-03, Guangdong, China).

Next, complementary DNA (cDNA) was generated utilizing SuperScript II Reverse Transcriptase, oligo 18dT, and random primers (hexamers) from Invitrogen. The qRT-PCR experiment was conducted using a Roche LightCycler 480 sequence detection system, employing the following conditions: an initial predenaturation step at 95 °C for 30 seconds, followed by 40 cycles of denaturation at 95 °C for 5 seconds, and annealing and extension at 60 °C for 30 seconds. The experimental approach employed involved the utilization of human glyceraldehyde-3-phosphate dehydrogenase (GAPDH) as the constitutive control. The quantification of gene expression levels was carried out utilizing the 2^-ΔΔ^ CT method. The primer sequences employed for qRT-PCR were as follows: CD276 sense, CTGGCTTTCGTGTGCTGGAGAA; and CD276 antisense, GCTGTCAGAGTGTTTCAGAGGC. HLA-E sense, CGGCTACTACAATCAGAGCGAG; HLA-E antisense, AATCCTTGCCGTCGTAGGCGAA. LGALS9 sense, CGTCAATGGCTCTGTGCAGCTGTC; and LGALS9 antisense, AGATCCACACTGAGAAGCTCTGGC. TNFRSF18 sense, CCAGTGTATCGACTGTGCCTCG; and TNFRSF18 antisense, CACAGCGTTGTGGGTCTTGTTC. PCR reactions for each sample were performed in triplicate. All results are presented as the mean ± standard deviation (SD).

### Analyses of the risk signature's clinical correlation, robust nomogram construction and evaluation

Using heatmaps and boxplots, the relationships between the risk score and clinical factors (age, grade, stage, T, N, M) were compared. In a stratified study, it was determined whether the risk score maintained its predictive potential across distinct age, stage, and grade subgroups. Independent risk variables were identified using univariate and multivariate Cox regression analyses (p < 0.05). The prognostic signature was then confirmed using the ICGC and GSE22541 (primary subgroup) databases using the same risk score calculation formula and statistical analysis methods (p < 0.05). The "rms" package in R was used to construct a prognostic nomogram, and the calibration curve was utilized to evaluate the nomogram's prediction ability. The concordance index was used to evaluate the predictive power of the signature using the bootstrap approach with 1,000 resamples. The predictive accuracy of the nomogram was assessed using time-dependent ROC curves. The predictive value between projected 3-, 5-, and 10-year survival events and actual observed outcomes was illustrated using nomogram calibration plots, and decision curve analysis (DCA) was utilized to evaluate the validity of the signature.

### Principal Component Analyses

We used principal component analysis (PCA) to minimize the dimensions and identify renal cancer patients with varying risk levels. For t-distributed stochastic neighbor embedding (t-SNE) analysis, the "Rtsne" package was used, while the prcomp function from the "stats" package was utilized for PCA.

### Immune status assessment

Using the single-sample gene set enrichment analysis (ssGSEA) technique, the scores of tumor microenvironment (TME) cells in each ccRCC sample were determined 9. Immunological and stromal scores for each patient were determined using the ESTIMATE method. The fractions of 22 human immune cell types in each TCGA ccRCC sample were determined 10. The expression levels of members of the human leukocyte antigen (HLA) family and immune checkpoint markers were compared between low- and high-risk groups. Several key TME immunosuppressive genes, including IL10, TGF-b, FOXP3, IL6, and FAP, were chosen to compare the expression level between the high-risk and low-risk groups using boxplots to elucidate the association between risk signature and TME immunosuppressive variables in ccRCC.

Tumor Immune Single-cell Hub (TISCH) collected data from GEO and ArrayExpress^11^ to create its scRNA-seq atlas, a TME-focused single-cell RNA-seq resource. TISCH2 (http://tisch.comp-genomics.org) has 190 datasets and 6,297,320 cells from cancer patients and healthy donors 12, allowing for the exploration of TME across several cancer types. Using TISCH2 datasets, we revealed the single-cell-level risk gene heterogeneity among immune cells in ccRCC.

### Forecasting immunotherapy response and validation of real-world cohorts

The immunophenoscore (IPS) analysis was performed randomly using machine learning by four classes of genes that determine immunogenicity: effector cells, suppressive cells, major histocompatibility complex molecules, and immunomodulators or checkpoints; its value increased as immunogenicity increased 13. The Cancer Immunome Atlas (TCIA) offered ccRCC patient IPSs (https://tcia.at/home). Immune Cell Abundance Identifier (ImmuCellAI) is a computer approach that was published in 2020 to predict the response to immune checkpoint inhibition based on the abundance of immune cells, especially various T cell subsets 14. Using the Tumor Immune Dysfunction and Exclusion (TIDE) algorithm (http:// tide.dfci.harvard.edu/), simulation experiments were conducted to comprehend the essential pathways involved with tumor immune evasion and to estimate the response potential of tumor immunotherapy.

In addition, two transcriptomic datasets containing clinical data from patients with advanced clear cell renal cell carcinoma treated with the anti-PD-1 agent Nivolumab (CheckMate025 cohort 15) and patients with advanced melanoma treated with various types of immunotherapy (TCGA-SKCM) were downloaded and analyzed to determine the response to immunotherapy and the predictive value of the risk score 16.

### Evaluation of the sensitivity of chemotherapeutic and molecular medicines

In order to predict the response to chemotherapy and molecular drugs, the "pRRophetic" package was used to generate the risk score; the half-maximal inhibitory concentration (IC50) was obtained using ridge regression between the low- and high-risk groups for 251 popular chemotherapeutic agents.

### Statistical analysis

R (version 4.2.0) and RStudio (version 2022.12.0+353 for macOS) were utilized to conduct statistical analyses. The Mann-Whitney U test was used to compare differences between two groups. For the study of differences between more than two groups, the Kruskal-Wallis test was utilized. The chi-square test was used to assess the difference in immune response frequency distributions. The Kaplan-Meier method was used to compare OS and disease-free survival (DFS) differences across groups. p<0.05 was deemed statistically significant in all two-tailed tests, unless otherwise noted.

## Results

### Identification of prognostic differentially expressed CICGs and subtype clustering

**Figure 2:**
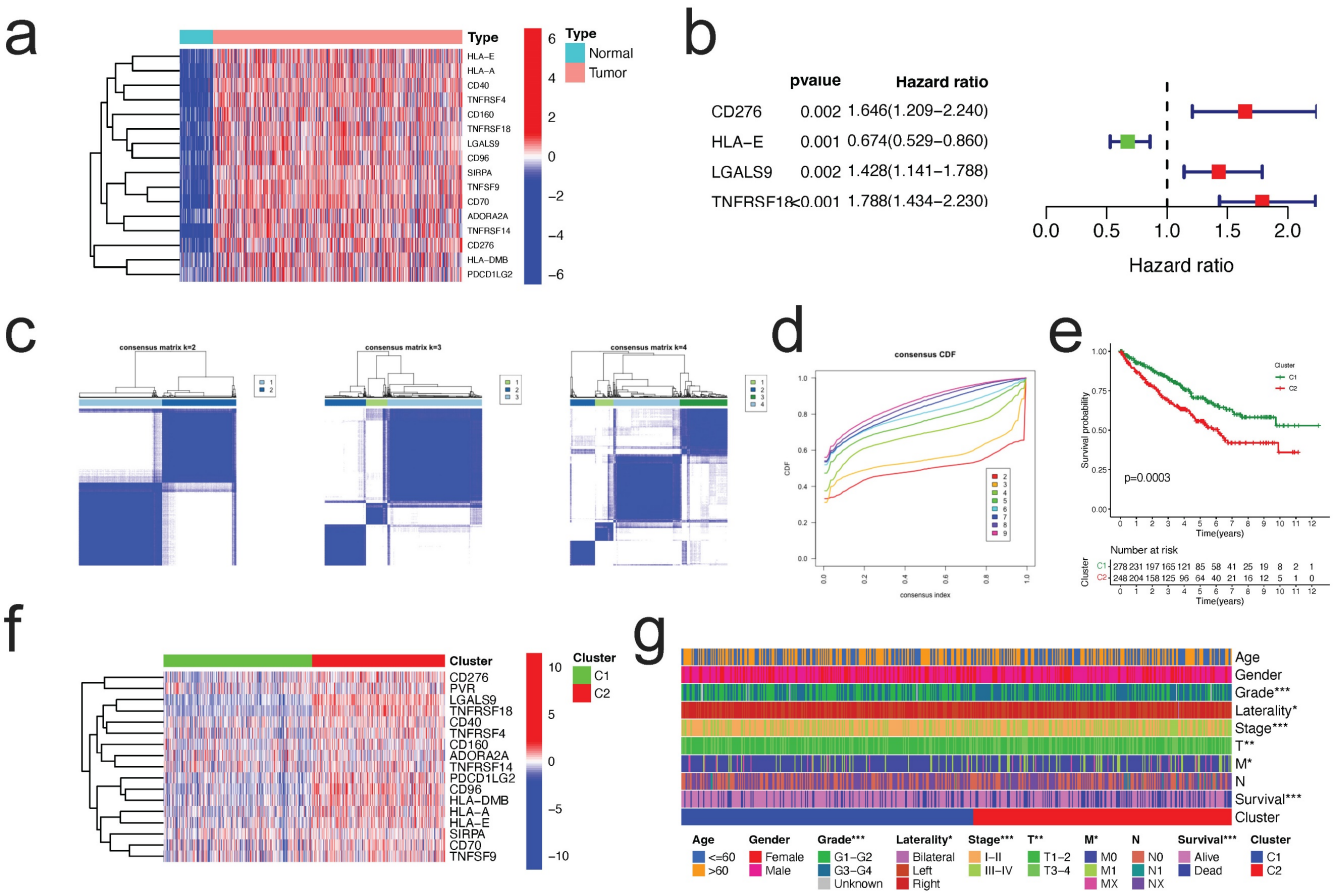
** Identification of prognostic differentially expressed CICGs and grouping of molecular subtypes. a.** Heatmap depicted the expression difference of CICGs between normal and tumor tissues in TCGA ccRCC cohort; **b.** Forest plot depicted the unicox results of 4 prognostic CICGs; **c.** Identification of molecular subtypes based on prognostic CICGs (showing k=2, 3, and 4); **d.** Consensus cumulative distribution function (CDF) curve for 2-9 curves; **e.** Kaplan-Meier curve for overall survival of all ccRCC patients with two cluster subtypes (log-rank test, p=0.0003); **f.** Heatmap displayed the expression difference of CICGs between Cluster C1 and C2 in TCGA ccRCC cohort; **g.** Heatmap illustrated the relationships between clinicopathologic features, and two cluster subtypes.

The expression of 17 ICGs was associated with that of CRGs (Table S2). A total of 16 ICGs exhibited differential expression between tumor and normal tissues (Table S3), all of these ICGs were highly elevated in tumor tissues (Figure 2a). Four CICGs, including CD276, HLA-E, LGALS9, and TNFRSF18, exhibited predictive significance in a Unicox analysis of these differentially expressed CICGs (Figure 2b).

To further investigate the expression of the four CICGs in ccRCC, we categorized patients with ccRCC using a consensus clustering algorithm (Figure 2c). Based on the clustering criteria (Figure 2d), we determined that k = 2 was ideal for sorting the entire cohort. Thus, two subgroups, named Clusters C1 and C2, were found, with Cluster C1 containing 278 cases and Cluster C2 containing 248 cases. Kaplan-Meier analysis of survival indicated that overall survival differed significantly between the two subtypes, and Cluster C2 had a significant survival disadvantage (log-rank test, p=0.0003, Figure 2e). The heatmap indicates that the expression of the 17 CICGs in the two clusters varied significantly (Figure 2f). In addition, the association between the two subtypes and a variety of clinicopathological parameters (survival status, age, gender, grade, laterality, stage, T, M, and N) was investigated, and the CICGs subtypes were linked to grade, laterality, stage, T, M, and survival status (Figure 2g).

### Determination of the DEGs between two clusters

**Figure 3:**
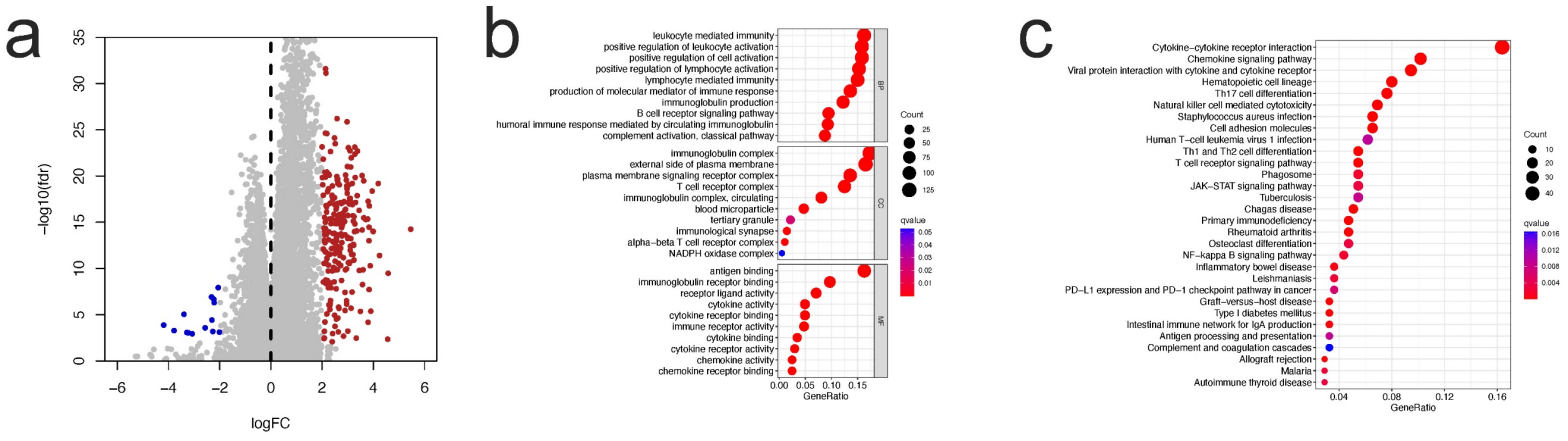
** identification of DEGs between two clusters and enrichment analysis. a.** volcano plot depicted the differentially expressed genes between two clusters (|logFC|>2 and p value <0.05); GO enrichment analysis **(b)** and KEGG enrichment analysis** (c)** of DEGs.

To investigate the probable biological role of each subtype of CICGs in ccRCC, the "limma" R program was used to determine the DEGs between the two clusters. The DEGs associated with the CICGs subtype were clustered on the volcano plot (Figure 3a). Gene ontology enrichment analysis revealed that a large number of immunological and cytokine-related processes were significantly enriched in the DEGs (Figure 3b, Table S4). Multiple immunological and cancer-related KEGG pathways were enriched, including cytokine interaction, chemokine signaling, Th17 cell differentiation, NF-kappa B signaling pathway, PD-L1 expression, and PD-1 checkpoint pathway in cancer. (Figure 3c, Table S5).

### Immune status difference between two clusters

**Figure 4:**
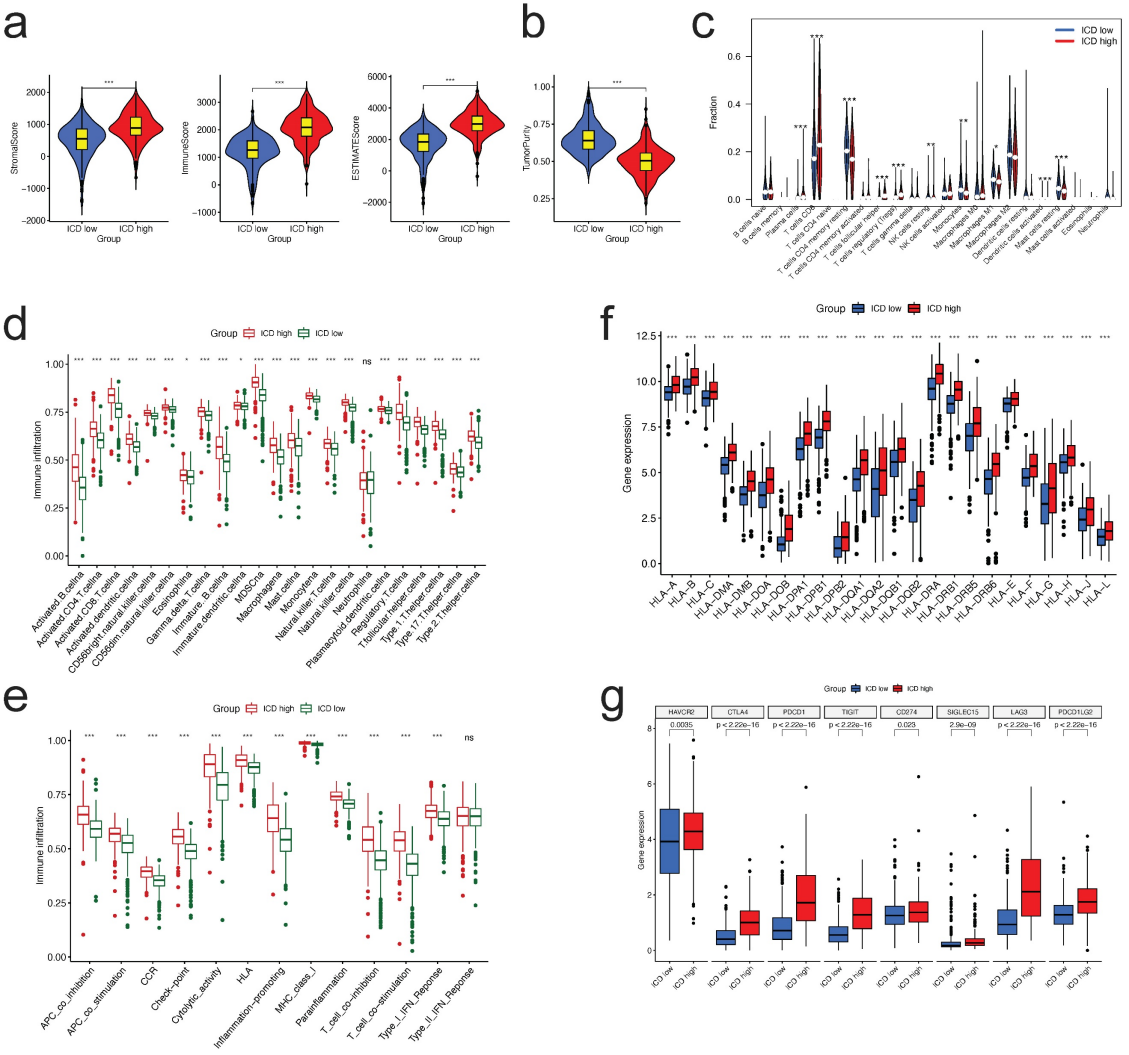
** Immune status difference between two clusters. a.** Correlations between different clusters and immune score, stromal score, and ESTIMATE score; **b.** Correlations between different clusters and tumor purity; **c.** Tumor immune cells difference between two clusters; **d,e.** Comparison of the ssGSEA scores for immune cells (d) and immune functions (e) for patients between the two clusters. The line in the box represents the median value. **f,g.** The expression of HLA family members (f) and immune checkpoints genes (g) between the two clusters.

To investigate variations in the composition of TME-infiltrating cells between the two clusters, we evaluated the TME score (stromal score, immune score, and estimate score) of the two subtypes using the ESTIMATE package. We designated C1 as the low immune checkpoint gene difference (ICD) group, and C2 as the ICD high group. The results indicated the lowest TME scores in the patients in the low ICD group (Figure 4a). Regarding tumor purity, the low ICD group had the highest score compared to the high ICD group (Figure 4b). Numerous immune cells, such as plasma cells, T cells CD8, T cells CD4 memory resting T cells, showed differences in expression between the two groups (Figure 4c).

We examined the enrichment score of immune cells and functions in the two groups using ssGSEA and found that Figure 7a, almost all immune cells and immune functions were suppressed in the ICD low group compared to the ICD high group (Figure 4d, e, respectively).

Considering the importance of immunotherapies based on HLA and checkpoint inhibitors, we analyzed the differences between the two ICD groups in terms of HLA family members and immune checkpoint expression. The majority of HLA members had high expression in the ICD high group (Figure 4f); with regard to checkpoints, we discovered that all these genes displayed differential expression between two groups, with CTLA4, PDCD1, and PD-L1 (CD274) demonstrating high expression in the ICD high group (Figure 4g).

### Prognostic risk signature construction and validation

To prevent overfitting of the model, LASSO analysis was performed on the four predictive CICGs in the TCGA cohort (Figure S1), which demonstrated that the four CICGs were independent prognostic markers for patients with ccRCC. To predict the OS of each ccRCC patient, a 4-CICGs signature (CD276, HLA-E, LGALS9, and TNFRSF18) was created. Table S6 presents the correlation coefficients.

**Figure 5:**
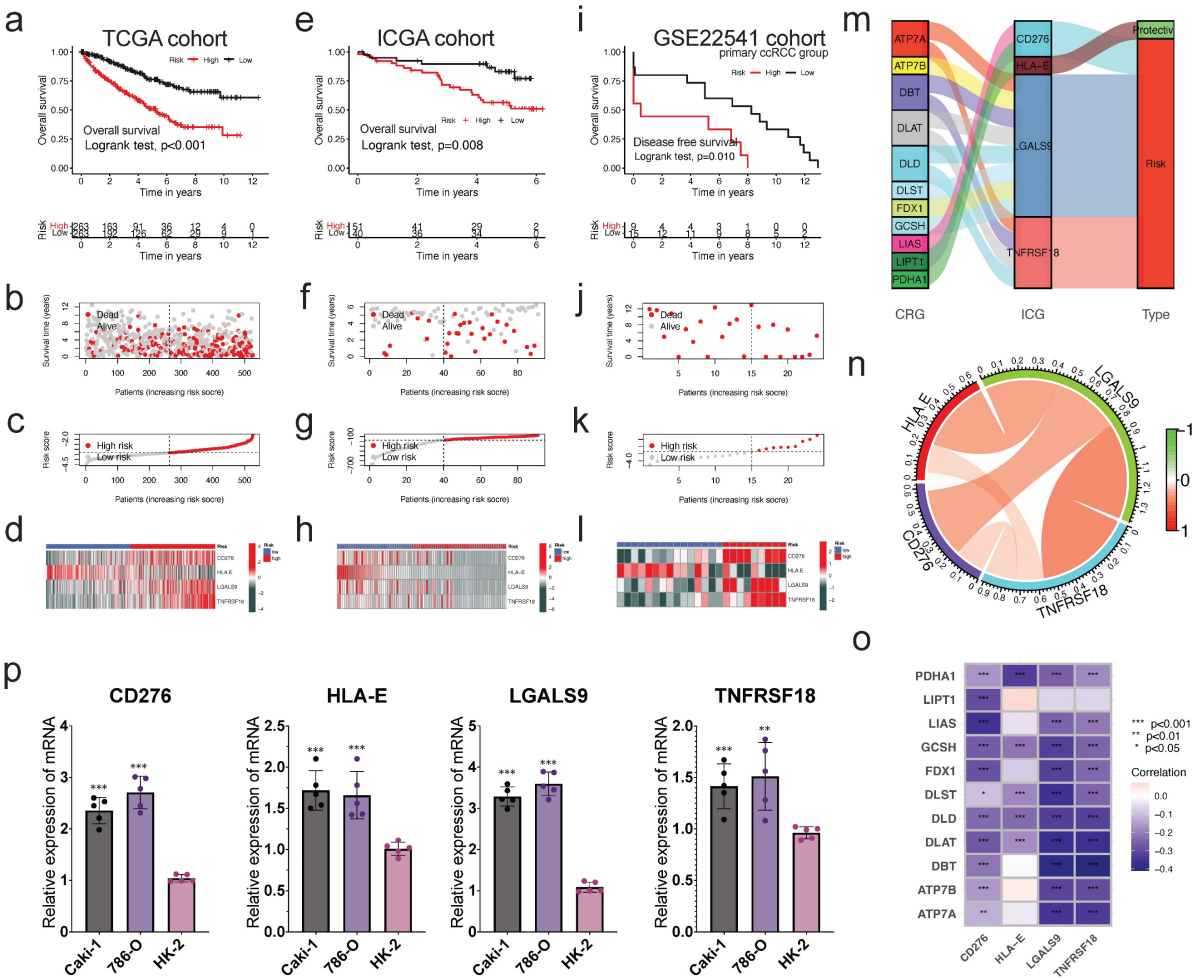
** Construction and validation of prognostic risk signature, and expression of CRGs.** The construction of risk signature in TCGA cohort **(a-d)**, validation in ICGA cohort **(e-h)** and primary ccRCC group in GSE22541 cohort** (i-l)**. Kaplan-Meier curves for OS of the patients from the high- and low-risk groups** (a,e,i)**; Scatter plot showing the correlation between the survival status and risk score of ccRCC patients **(b,f,j)**; Risk score distribution plot showing the distribution of high-risk and low-risk ccRCC patients **(c,g,k)**; Heatmap showing expression of the for CICGs between the high- and low-risk groups **(d,h,l)**. **m.** Sankey diagram showing the degree of connection between the CRGs, ICG, and risk signature type (protective or risk); **n.** The co-relationship of four risk genes in the signature; **o.** Heatmap showing the co-expression relationship of cuproptosis-related genes and four signature genes; **p.** The expression levels of four risk genes in vitro by qRT-PCR. * P < 0.05, ** P < 0.01, *** P < 0.001.

The patients were classified into high- and low-risk groups based on the median risk score. Kaplan-Meier curves indicated that the high-risk group in TCGA cohort had a worse prognosis (Figure 5a). The scatter plot suggested that ccRCC patients with a high-risk score had a poorer survival rate than those with a low-risk score (Figure 5b), and the risk-score distribution map was consistent with the classification of patient groups (Figure 5c). The risk gene expression heatmap revealed remarkable expression disparities between the high- and low-risk groups for the four CICGs (Figure 5d).

To externally validate the predictive ability of the risk signature, the risk scores of patients in the ICGC and GSE22541 cohorts (24 primary ccRCC patients) were computed. Kaplan-Meier survival analysis of OS in the ICGC cohort showed that the outcome was comparable to that of the TCGA cohort (Figure 5e). Concerning GSE22541, survival analysis revealed that the high-risk group had a significantly worse DFS than the low-risk group (Figure 5i). Similarly, in the ICGC and GSE22541 cohorts, the high-risk group had a lower survival rate than the low-risk group, and the risk-score distribution map verified that the high-risk group had a higher risk score (Figure 5fg, jk, respectively). The heatmaps illustrate consistent expression patterns of the four risk CICGs in the two cohorts (Figure 5h, l, respectively).

Eleven cuproptosis-related genes were linked to four CICGs. The sankey graphic reveals a broad and intricate relationship between them (Figure 5m). According to the correlation analysis, the four CICGs were positively associated with each other (Figure 5n). When we plotted the correlation between the four CICGs and 11 CRGs, we found that the majority were negatively associated (Figure 5o).

Finally, the expression levels of four CICGs were verified by qRT- PCR in the normal and tumor cells (Figure 5p). The results showed that the overall trend in the expression levels of all CICGs increased obviously in ccRCC cell lines (Caki-1, and 786-O) compared with normal renal proximal tubule epithelial cells (HK-2), which are consistent with our previous bioinformatics analysis based on public database.

### PCA, Stratified Survival Analysis and correlation between the risk score and Clinicopathological Characteristics

**Figure 6:**
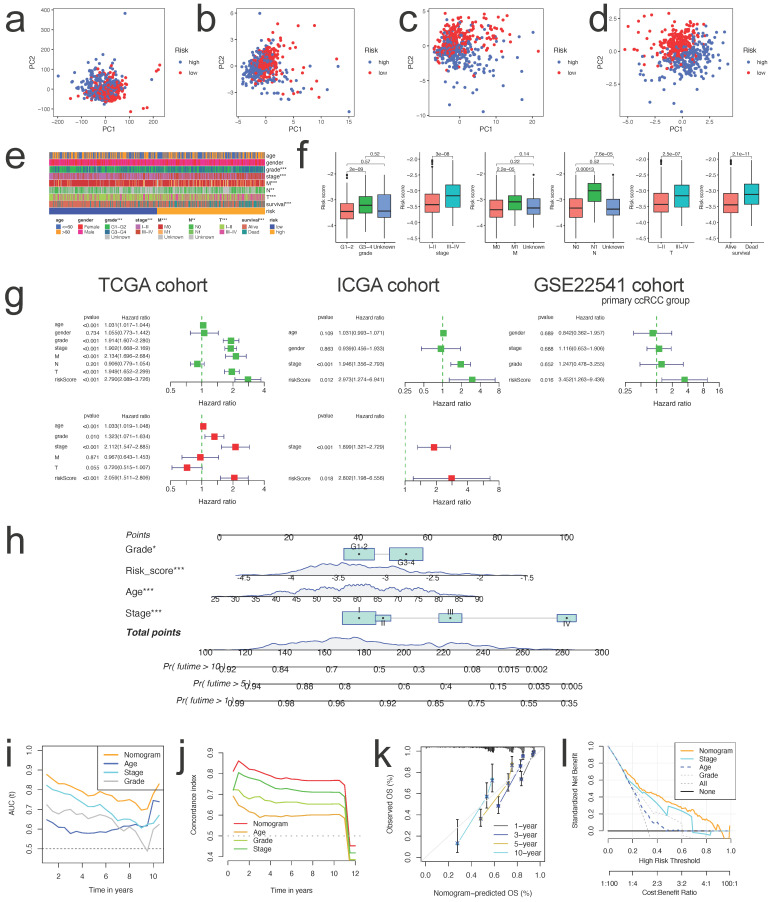
** PCA analysis, nomogram construction and evaluation.** PCA analysis for the entire gene expression** (a)**, expression of CRGs **(b)**, expression of ICGs **(c)**, and expression of four risk CICGs** (d)** in high and low risk groups in ccRCC patients; **e.** Heatmap showing the prognostic signature and clinicopathological features of the low- and high-risk patients with ccRCC; **f.** Correlation analyses of the prognostic signature with the clinicopathological characteristics of the patients with ccRCC according to the grade, stage, M, N, T, and survival status, respectively (all P<0.01); **g.** Univariate and multivariate Cox survival analysis showed that risk signature was an independent prognostic factor in three cohorts, respectively (TCGA, ICGC, and GSE22541); **h.** Prognostic nomogram for predicting 1-, 5-, and 10-year OS of patients with ccRCC; **i,j.** Time-dependent ROC curves (i) and C-index curves (j) to compare AUC values of the nomograms and other clinical factors within a range of time; **k.** Calibration curves of nomogram displayed the concordance between predicted and observed 1-, 3-, 5-, and 10-year OS; **l.** DCA curves for the nomogram and stage, age, grade.

The PCA schematic diagram depicts two distinct risk categories for ccRCC patients based on total gene expression, expression of cuproptosis genes, expression of genes associated to immunological checkpoints, and expression of four risk CICGs (Figure 6a-d, respectively).

We performed stratified survival analysis of clinicopathological factors including age (<=60 years vs. >60 years), gender (Male vs. Female), grade (Grade 1-2 vs. Grade 3-4), stage (Stage I-II vs. Stage III-IV), T (T1-2 vs. T3-4), M (M1 vs. M0), and N (N1 vs. N0) to evaluate the predictive ability and stability. The findings of Kaplan-Meier survival analysis encompassing diverse clinical characteristics further demonstrated that OS in high-risk group was poorer than that in low-risk group (p< 0.01, except patients in N1) (Figures S2).

There were strong connections between the risk scores and the aforementioned clinicopathological parameters of ccRCC (Figure 6e), i.e., as the stage, grade, metastasis, and mortality increased, so did the risk score (Figure 6f, all p<0.001).

### Constructing and Assessing the Nomogram

To examine if the risk score was an independent prognostic factor in patients with ccRCC, univariate and multivariate Cox regression analyses were conducted utilizing the patients' clinical features and risk scores. This was confirmed in TCGA, ICGC, and GSE22541 cohorts (Figure 6g). The nomogram was then created utilizing the independent prognostic parameters to predict the 1-, 5-, and 10-year OS of patients with ccRCC (Figure 6h). The time-dependent AUC analysis revealed that the nomogram's predictive value was significantly greater than that of age, stage, and grade from 1 to 10 years (Figure 6i). The C-index plot demonstrated the same trend (Figure 6j). We utilized the calibration curve to determine if the actual prognosis value matched the projected value of the nomogram and found that the 1-, 3-, 5-, and 10-year survival rate calibration curves were compatible with the nomogram (Figure 6k). The DCA curves also demonstrated that the nomogram had a positive predictive effect and greater clinical value than stage, age, and grade (Figure 6l).

### Correlation between the risk score and tumor infiltrating immune cells

**Figure 7:**
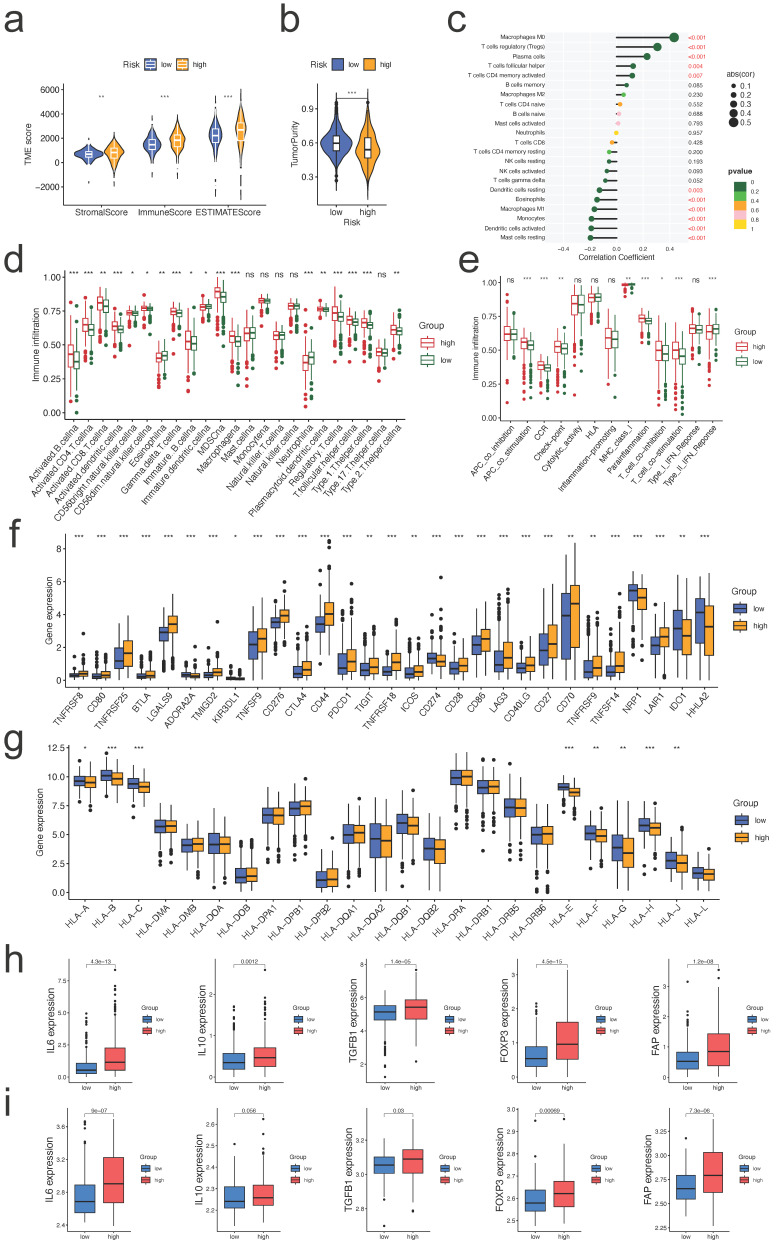
** Correlation between the risk signature and the immune microenvironment. a.** Correlations between risk group and immune score, stromal score, and ESTIMATE score; **b.** Correlations between risk groups and tumor purity; **c.** Correlations between risk score and immune cell type based on CIBERSORT; **d,e.** Comparison of the ssGSEA scores for immune cells (d) and immune functions (e) for patients between the high- and low-risk groups; **f,g.** The expression of immune checkpoints genes (f) and HLA family members (g) between the high- and low-risk groups; **h,i.** Expression of TME immunosuppressive cytokines and markers in TCGA (h) and GSE73731 (i) cohorts.

Comparing the TME scores of each risk score group, we discovered that the group with the high-risk score had significantly higher TME scores and reduced tumor purity (all p<0.01, Figure 7a, b, respectively). Risk score was correlated positively with Macrophages M0, T cells regulatory, plasma cells, T cells follicular helper, and T cells CD4 memory activated, and negatively with mast cells resting, dendritic cells activated, monocytes, macrophages M1, eosinophils, and dendritic cells activated (all p<0.05, Figure 7c). Many immune cells (Figure 7d) and immunological functions (Figure 7e) displayed significant variations between the two risk groups, with the high-risk group having a larger number of activated immune cells and immunological functions. Given the significance of checkpoint inhibitor-based immunotherapies, we investigated further the variations in immune checkpoint expression between the two groups. There were significant variations in the expression of CD44, CD276, CTLA4, PDCD1, CD274, LGALS9, and TNFSF14 between the two groups of patients (Figure 7f, all p<0.05). Similar outcomes were also observed in two additional validation datasets (GSE22541, GSE73731, Figure S3). As for the expression difference of HLA members between two groups, we observed that the expression of HLA-A/B/C/E/F/G/H/J was significantly different (Figure 7g, all p<0.05). Similar outcomes were also observed in two more validation sets (Figure S4). These findings suggest that our risk score may have a correlate with the patient's responsiveness to medicines targeting the aforementioned checkpoints.

As a result, we continued to investigate the relationship between the risk signature and the expression level of TME immunosuppressive factors, including the key cytokines of immunosuppressive TME: cancer-associated adipocytes activated marker IL-6 17, IL-10 and TGF-b 18, Treg marker FOXP3 19, and CAF marker FAP 20. In the high-risk group of the TCGA cohort, the expression levels of the five cytokines and markers listed above were elevated (Figure 7h). Except for IL10, a similar trend was seen in the external validation dataset (GSE73731) (Figure 7i). Collectively, the aforementioned findings suggested that ccRCC patients with a high-risk score may have a strong immunosuppressive TME, which led to the immune evasion of tumor cells and the poor prognosis.

### Single-Cell level analysis of risk signature

**Figure 8:**
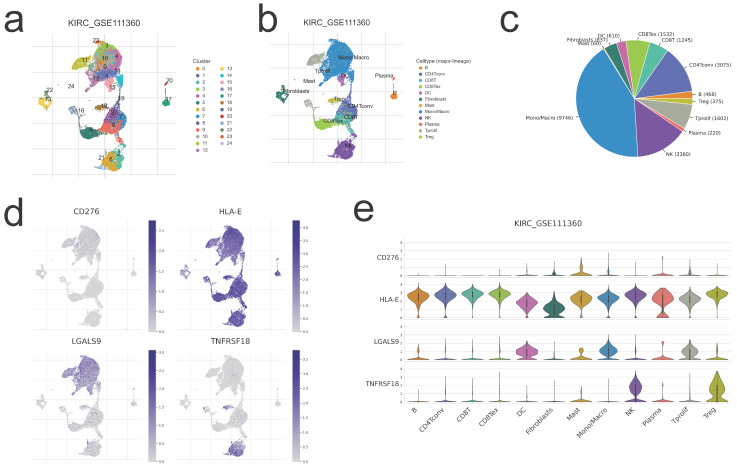
** Single-cell level evaluation of risk genes by TISCH2 database. a,b,c.** The cell types and their subgroup distribution in KIRC GSE111360dataset; **d.** Distribution of four risk genes in different cells in KIRC GSE111360 dataset; **e.** Distribution of four risk genes' expression in different cell types using violin plot in KIRC GSE111360 dataset.

Herein, we intended to localize four risk genes at the level of a single cell in order to investigate their probable relationship with immune cells. By examining the TISCH2 database, we determined that all four genes were expressed in subpopulations of immune single cells. 24 clusters (Figure 8a) and 12 cell types (Figure 8b) were detected in KIRC GSE111360, including CD4 T conventional, CD8 T cell, monocyte/macrophage cell, dendritic cell, natural killer cell, Treg cells, etc., with monocyte/macrophage cells exhibiting the highest cell numbers (Figure 8c). And all of the 12 cell types exhibited four risk gene expression (Figure 8d,e), with the mast cell subgroup cells displaying the greatest abundance of CD276; CD4 T conventional cell, CD8 T cell, as well as natural killing cell subgroups showed the obviously more abundance of HLA-E; as to LGALS9 and TNFRSF18, the abundance subgroup cells are mono/macrophage cell, and natural killing cell, respectively (Figure 8e). The preceding single-cell analyses indicated that the risk genes were strongly expressed in all immune cell subsets of ccRCC, hence corroborating the association between risk score and TME.

### Estimating immunotherapeutic advantages

**Figure 9:**
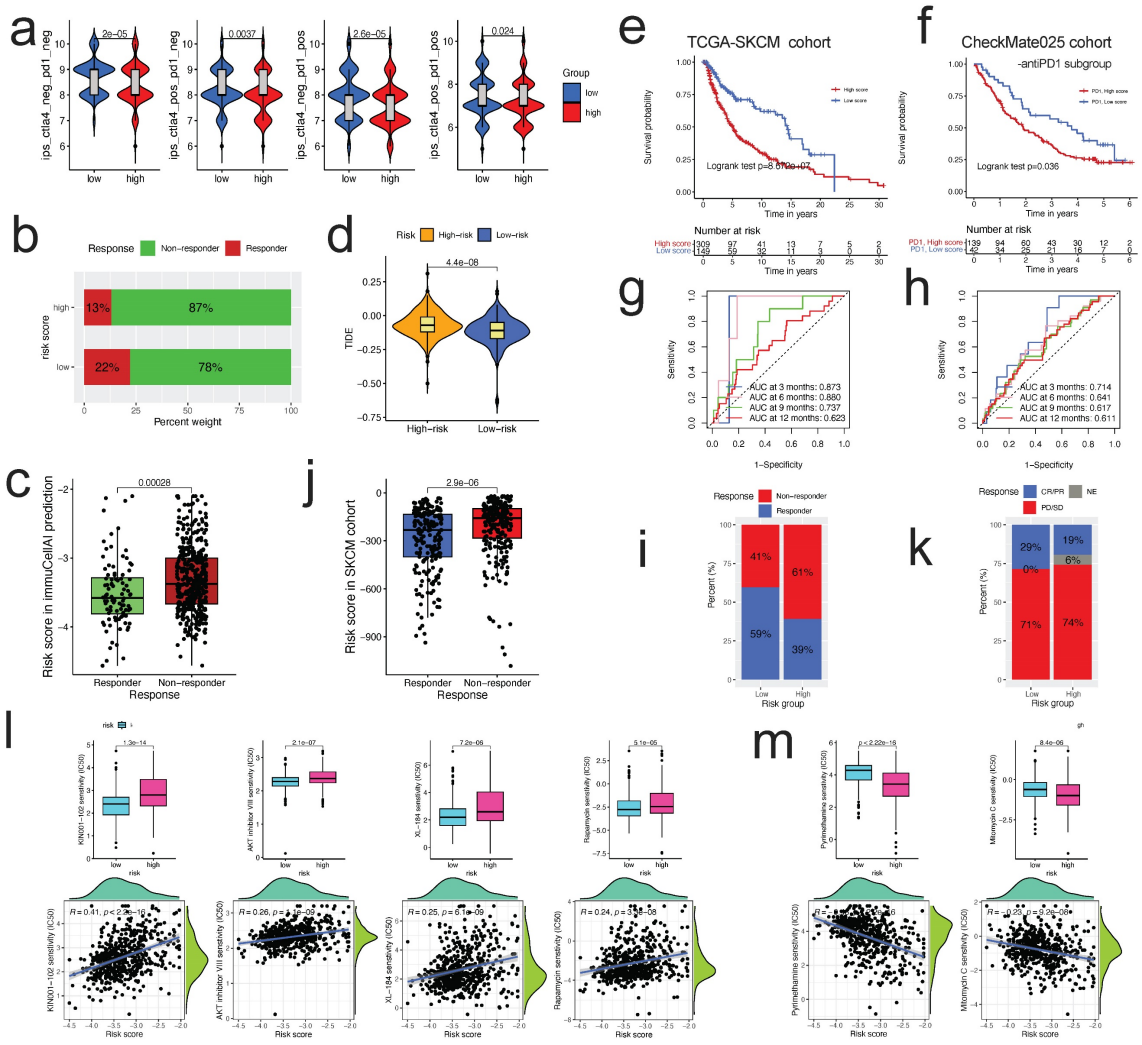
** The risk score could predict patients' immunotherapeutic benefit. a.** The association between IPS and the risk score in ccRCC patients of TCGA cohort; **b.** The differences in response results to immunotherapy between low-risk and high-risk groups by ImmuCellAI algorithm; **c.** The scatter plot shows the correlation between immunotherapy responsiveness and risk score in ccRCC patients by ImmuCellAI algorithm; **d.** TIDE score difference in high and low risk groups; **e, f.** K-M survival analysis of the risk subgroups in TCGA SKCM cohort(e), and CheckMate025 cohort (anti-PD1 subgroup) (f); **g, h.** ROC curves and their AUC values for risk score in TCGA SKCM cohort(g), and CheckMate025 cohort (anti-PD1 subgroup) (h); **i, k.** Proportions of anti-multiple immunotherapy response (l) and anti-PD1 immunotherapy response (k) in high and low risk groups in TCGA SKCM cohort, and CheckMate025 cohort (anti-PD1 subgroup); **j.** The scatter plot shows the correlation between immunotherapy responsiveness and risk score in SKCM cohort; **l, m.** The half-maximal inhibitory concentration (IC50) of drugs for targeted therapy and chemotherapy in high- and low-risk groups in the TCGA cohort. The corresponding linear correlation plots between risk score and drugs were showed below.

Using Immunophenoscore, we subsequently assessed the responsiveness of subgroups stratified by risk score to immune checkpoint inhibitors. The low-risk group had a higher IPS, indicating stronger immunogenicity of tumors and greater sensitivity to ICI, as demonstrated by our findings (Figure 9a). Using the ImmuCellAI and TIDE algorithms, we assessed the potential immunotherapy response of each patient. Patients in the low-risk group were more likely to react to immune checkpoint blockade than those in the high-risk group (13% vs. 22%) (Figure 9b). Respondents had a lower risk score than non-respondents (p<0.001; Figure 9c). Patients in the low-risk group had lower TIDE scores, indicating a decreased possibility of immune evasion. The lower the TIDE score, the lower the likelihood of immune evasion and the greater the likelihood that the patient will benefit from ICI therapy (Figure 9d). Collectively, these data suggest that the predictive risk signature may predict the potential response to immunotherapy in patients with ccRCC; individuals in the lower-risk group had more prospective immunotherapy benefits.

We investigated the predictive value of the risk score in 30 distinct TCGA cancer cohorts containing 9397 tumors (Table S7). Except for ccRCC, the risk score was validated as a favorable prognostic biomarker in six independent TCGA cohorts (Figure S5), including mesothelioma, adrenocortical carcinoma, liver hepatocellular carcinoma, head and neck squamous cell carcinoma, stomach adenocarcinoma, and kidney renal chromophobe.

Monoclonal antibodies that suppress the T-cell inhibitory molecules PD-L1 and PD-1 have emerged as cancer therapies with extraordinary and synergistic survival benefits. Next, we examined the predictive value of the risk score for immune checkpoint treatment in the real world by classifying patients in the TCGA SKCM and Checkmate025 cohorts (anti-PD1 subgroup) into high- and low-risk categories. In the TCGA SKCM (Figure 9e) and Checkmate025 cohorts (Figure 9f), patients with a high-risk score had a shorter OS than those with a low-risk score. In TCGA SKCM and Checkmate025 cohorts, the predictive significance of the risk score for checkpoint immunotherapy was also verified (Figure 9g, h, respectively). We evaluated the clinical applicability of risk score in response to immunotherapy. Patients in the low-risk group were more likely to benefit from immune checkpoint therapy (TCGA SKCM cohort, two-sided, p<0.0001, Figure 9i). The risk score was lower in respondents than in non-responders (p<0.0001; Figure 9j). Although the difference was not statistically significant, the Checkmate025 cohort showed a similar general tendency (Figure 9k).

### Chemotherapeutic and molecular drug sensitivity prediction

Using the "pRRophetic" R package, we studied the association between the risk score and sensitivity to chemotherapy and targeted therapy medicines in patients with ccRCC. The calculated IC50 values of 63 distinct medications varied considerably between the two risk groupings (all p<0.01, Table S8), as shown by our findings. 42 drugs were more sensitive in the low-risk group than in the high-risk group, such as p38 MAPK inhibitor (KIN001-102), AKT inhibitor VIII, mTOR inhibitor (Rapamycin), and VEGFR2 inhibitor (XL-184) (Figure 9l); 21 drugs were more sensitive in the high-risk group than in the low-risk group, such as DHFR inhibitor (Pyrimethamine), Mitomycin C, and VEGFR2 inhibitor (XL-184) (Figure 9m). These results indicated that the risk score might be utilized to predict chemotherapy and targeted treatment.

## Discussion

In normal situations, the immune system of the host, specifically cytotoxic T lymphocytes (CTLs) and natural killer (NK) cells, possess the ability to identify and eliminate malignant cells 21. However, the immune response is intricately regulated by various activating and inhibitory mechanisms in order to prevent the occurrence of autoimmune events and maintain a balanced state of immunological dynamics. The activation of the immunological checkpoint (IC) receptors, which are present on cytotoxic T lymphocytes (CTL) and natural killer (NK) cells, occurs when they interact with IC ligands expressed on tumor cells or immunosuppressive cells. This interaction initiates the activation of the IC signaling pathway, which serves as the fundamental mechanism regulating the immune response. Currently, the tumor exhibits immunological evasion by inhibiting cytotoxicity and immune surveillance 22. Furthermore, the presence of tumors might impede the immune response against them by increasing the expression of immune checkpoints (ICs), leading to the development of an immunosuppressive tumor microenvironment 23. Tsvetkov et al. (7) have elucidated a novel kind of cell death, known as cuproptosis, by effectively utilizing the pathophysiological importance of copper. This discovery holds promise for the development of a novel approach to anticancer therapy. The phenomenon of cuproptosis-induced cell death has attracted significant attention in the field of science in recent years. However, there is a limited amount of research that has investigated the interplay between cuproptosis, prognosis, and the immune response against tumors in ccRCC. In order to investigate the correlation between gene expression profiles, prognosis, and antitumor immunity in ccRCC, a prognostic risk signature was constructed. This signature was based on four cuproptosis-related ICGs. The data utilized for this work was obtained from The Cancer Genome Atlas (TCGA), International Cancer Genome Consortium (ICGC), and Gene Expression Omnibus (GEO) databases. It was established that the risk signature exhibited characteristics of an autonomous risk factor for overall survival (OS), leading to the development and evaluation of a resilient nomogram. The study conducted a comprehensive analysis of the tumor microenvironment (TME), comparing various aspects such as the TME's overall characteristics, specific genes related to disease progression (checkpoint genes), members of the HLA gene family, and immunosuppressive cytokines and markers present in the TME. This comparison was performed across different risk groups. Additionally, the study evaluated the sensitivity of patients with different expression signatures to immunotherapy in real-world settings, specifically focusing on patients from the SKCM and Checkmate025 cohorts. The aim was to gain a deeper understanding of the variations in anti-tumor immune responses and to assess the ability to predict patient response to immunotherapy.

Renal cell carcinoma (RCC) is commonly recognized as a tumor with immunogenic properties. However, it has been observed that RCC can trigger the recruitment of immune-suppressive cells, such as regulatory T cells and myeloid-derived suppressor cells, into the tumor microenvironment (TME), leading to the impairment of immune responses. Numerous hypothetical processes have been postulated to elucidate the manner in which these diverse cell types entering tumors hinder the establishment of a potent immune response against the tumor. These mechanisms encompass the suppression of effector T cell and antigen-presenting cell function by the overexpression of inhibitory factors, such as checkpoint molecules. The use of immune checkpoint inhibitors (ICIs) that specifically target the PD-1/PD-L1 pathway and cytotoxic CTLA-4 has brought about a significant shift in the therapeutic approach for RCC 24. At present, the typical therapeutic approach entails administering this medication to individuals diagnosed with advanced RCC. In comparison to most other types of solid tumors that respond to anti-PD-1 treatment, the presence of a significant number of CD8+ T cells in ccRCC patients has previously been linked to a poorer prognosis 15, 25. Hence, it is imperative to establish dependable biomarkers that can accurately assess the efficacy of checkpoint blockade treatments in order to optimize their therapeutic impact 24. The patients classified as high-risk exhibited a bleak prognosis, while the application of TIDE and ImmuCellAI in predicting immunotherapy response indicated potential benefits for individuals categorized as low-risk. Likewise, a thorough examination of cohorts in real-world settings has confirmed the enduring predictive capability of the risk signature.

The reaction to immune checkpoint inhibitors (ICIs) is contingent upon the interplay between tumor cells, immune cells, and other immunomodulatory factors within the tumor microenvironment. Previous studies have identified regulatory T cells (Tregs), Kupffer cells, as well as monocyte- and myeloid-derived macrophages as the primary cellular components accountable for inducing this immunosuppressive response. These mechanisms encompass the secretion of immunosuppressive cytokines, such as interleukin (IL)-10, as well as the mobilization of regulatory T cells (Treg cells) and CD4+ T helper 17 (Th17) cells 24. Previous studies have provided evidence that Th2 cells, which mostly secrete IL-2 and IL-10, play a role in promoting immunosuppression, tumor formation, and metastasis 26. The findings of our analysis indicate a significant association between M0, Tregs, CD4 cells, T follicular helper cells, and the risk score, suggesting the presence of immunosuppression in persons belonging to high-risk categories. This observation highlights the necessity for a more focused examination of this specific demographic.

As previously noted by researchers (27), there is a crucial need to conduct a more comprehensive examination of the mutual regulatory interaction between CRG and ICG, as it serves as the foundation for our effective therapeutic advancement. Recent research has introduced a novel algorithm known as the single-cell multi omics gene co-regulation algorithm. Additionally, the advancement of single-cell multi-omics technology has shown great potential 28. The method presented in this study demonstrates a high level of efficiency in identifying co-regulatory programs, which can be utilized to elucidate molecular pathways and ascertain precise targets. Anticipation is held for forthcoming presentations of comprehensive studies that center on CRG and ICG. Additionally, additional exploration and enhancement of novel technologies are expected to foster our progress.

Notwithstanding the aforementioned, this study exhibits a number of shortcomings. This study focused solely on the examination of CRGs and ICGs, while not considering additional biomarkers associated with the immune system. Furthermore, there is significant variation in the abundance of immune cells among individuals, making it difficult to ascertain the extent to which gene expression levels are primarily influenced by the specific kind of immune cell. Despite conducting real-world validation for our risk signature in terms of prognosis and immunotherapy response, it is imperative to conduct extensive clinical sample studies in order to validate the prediction efficacy of the model.

## Conclusion

In summary, a novel gene signature associated with cuproptosis-related immunological checkpoints was formulated to predict the prognosis of patients with ccRCC and to characterize the immune microenvironment. Anticipating immunotherapy responses is also feasible in patients. The aforementioned findings have the potential to make a valuable contribution towards the advancement of personalized treatment strategies for individuals diagnosed with ccRCC. Furthermore, it facilitates a more comprehensive comprehension of the significance of cuproptosis in determining patient prognosis and the development of antitumor immunity in individuals diagnosed with ccRCC.

## Supplementary Material

Supplementary figures.

Supplementary tables.

## Abbreviations

ccRCC: clear cell renal cell carcinoma
CRGs: cuproptosis-related genes
ICGs: immune-checkpoint genes
CICGs: cuproptosis-related immune-checkpoint genes
OS: overall survival
DFS: disease-free survival
DEGs: differentially expressed genes
LASSO: least absolute shrinkage and selection operator
AUC: area under the time-dependent ROC curve
PCA: principal component analysis
TME: tumor microenvironment
ICI: immune checkpoint inhibitor
PD1: programmed death 1
PD-L1: programmed death ligand 1
CTLA4: CTL associated protein 4
IPS: immunophenoscore
