# Supplementary material for: Prognosis, Immune Microenvironment Infiltration and Immunotherapy Response in Clear Cell Renal Cell Carcinoma Based on Cuproptosis-related Immune Checkpoint Gene Signature
Source: J Cancer. 2023 Oct 9;14(17):3335–50. doi: 10.7150/jca.88467 (PMC10622984; doi:10.7150/jca.88467)

Figure S1. LASSO profile.
a. LASSO coefficient profiles of 4 genes for survival; b. LASSO analysis identified 4 genes for survival.

Figure S2. Survival analysis of ccRCC patients stratified by different clinicopathological features.

Figure S3. The expression of immune checkpoints genes between the high- and low-risk groups in external cohorts.
Figure S4. The expression of HLA family member genes between the high- and low-risk groups in external cohorts.
Figure S5. Subgroup analyses estimating prognostic value of risk score in different cancer types from TCGA data sets.
The length of horizontal line represents the 95% confidence interval for each group. The vertical box line represents the HR of all patients.

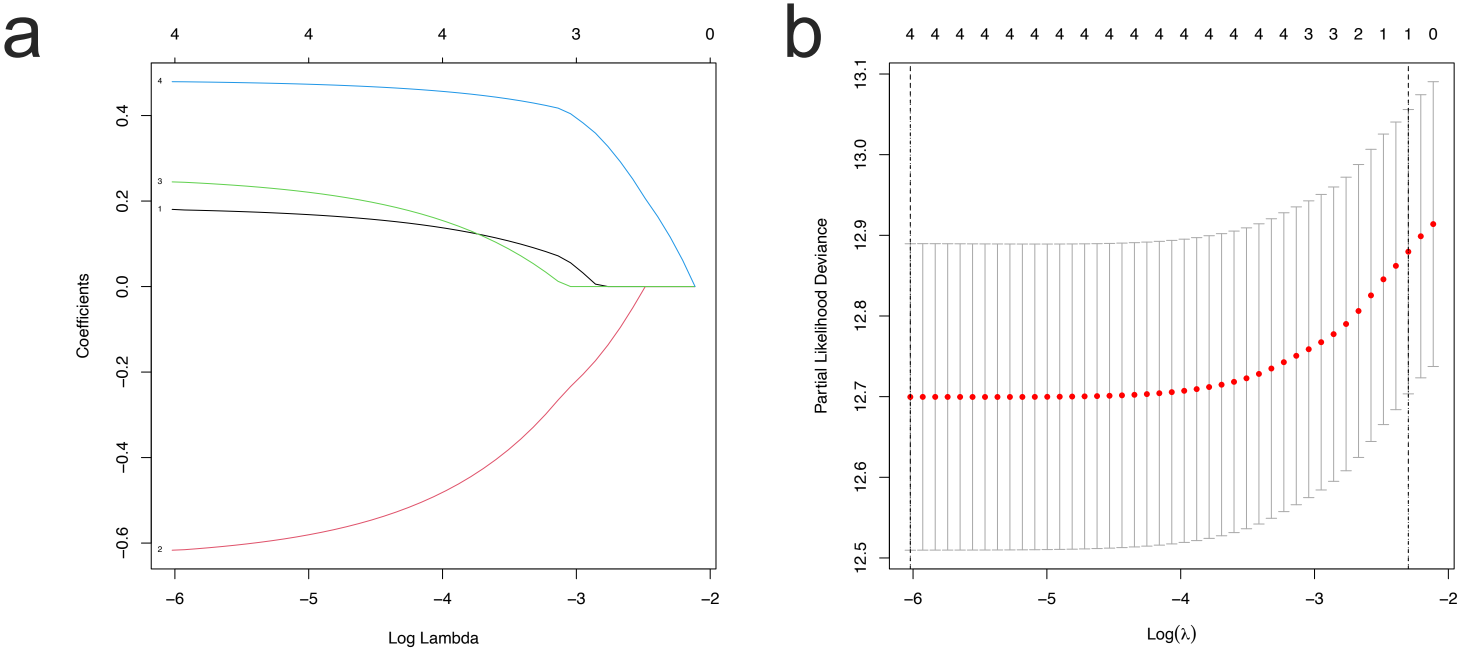

a
4 4 4 3 0
0.4
0.2
0.0
−0.2
−0.4
−0.6
Coefficients
−6 −5 −4 −3 −2
Log Lambda
b
4 4 4 4 4 4 4 4 4 4 4 4 4 4 4 4 3 3 2 1 1 0
13.1
13.0
12.9
12.8
12.7
12.6
12.5
Partial Likelihood Deviance
−6 −5 −4 −3 −2
Log(λ)

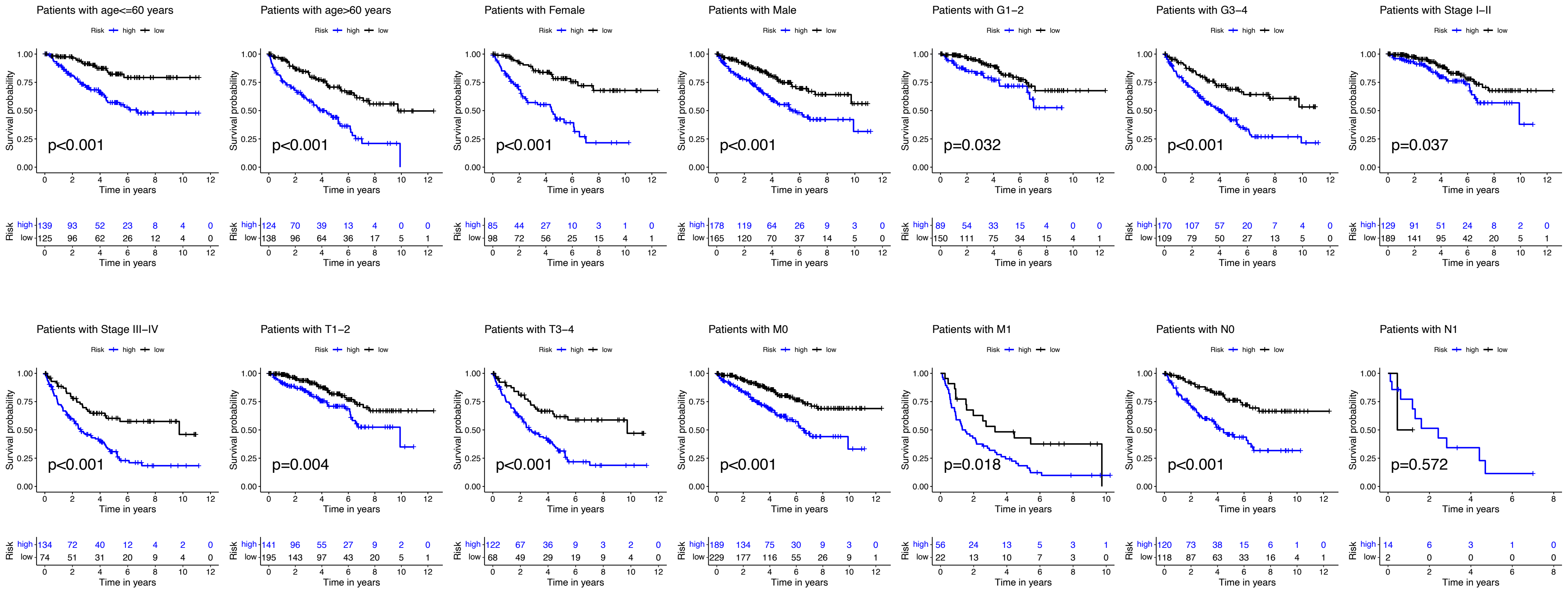

Patients with age<=60 years
p<0.001
high 139 93 52 23 8 4 0
low 125 96 62 26 12 4 0
Patients with age>60 years
p<0.001
high 124 70 39 13 4 0 0
low 138 96 64 36 17 5 1
Patients with Female
p<0.001
high 85 44 27 10 3 1 0
low 98 72 56 25 15 4 1
Patients with Male
p<0.001
high 178 119 64 26 9 3 0
low 165 120 70 37 14 5 0
Patients with G1−2
p=0.032
high 89 54 33 15 4 0 0
low 150 111 75 34 15 4 1
Patients with G3−4
p<0.001
high 170 107 57 20 7 4 0
low 109 79 50 27 13 5 0
Patients with Stage I−II
p=0.037
high 129 91 51 24 8 2 0
low 189 141 95 42 20 5 1
Patients with Stage III−IV
p<0.001
high 134 72 40 12 4 2 0
low 74 51 31 20 9 4 0
Patients with T1−2
p=0.004
high 141 96 55 27 9 2 0
low 195 143 97 43 20 5 1
Patients with T3−4
p<0.001
high 122 67 36 9 3 2 0
low 68 49 29 19 9 4 0
Patients with M0
p<0.001
high 189 134 75 30 9 3 0
low 229 177 116 55 26 9 1
Patients with M1
p=0.018
high 56 24 13 5 3 1
low 22 13 10 7 3 0
Patients with N0
p<0.001
high 120 73 38 15 6 1 0
low 118 87 63 33 16 4 1
Patients with N1
p=0.572
high 14 6 3 1 0
low 2 0 0 0 0
Risk high low
Survival probability
Time in years

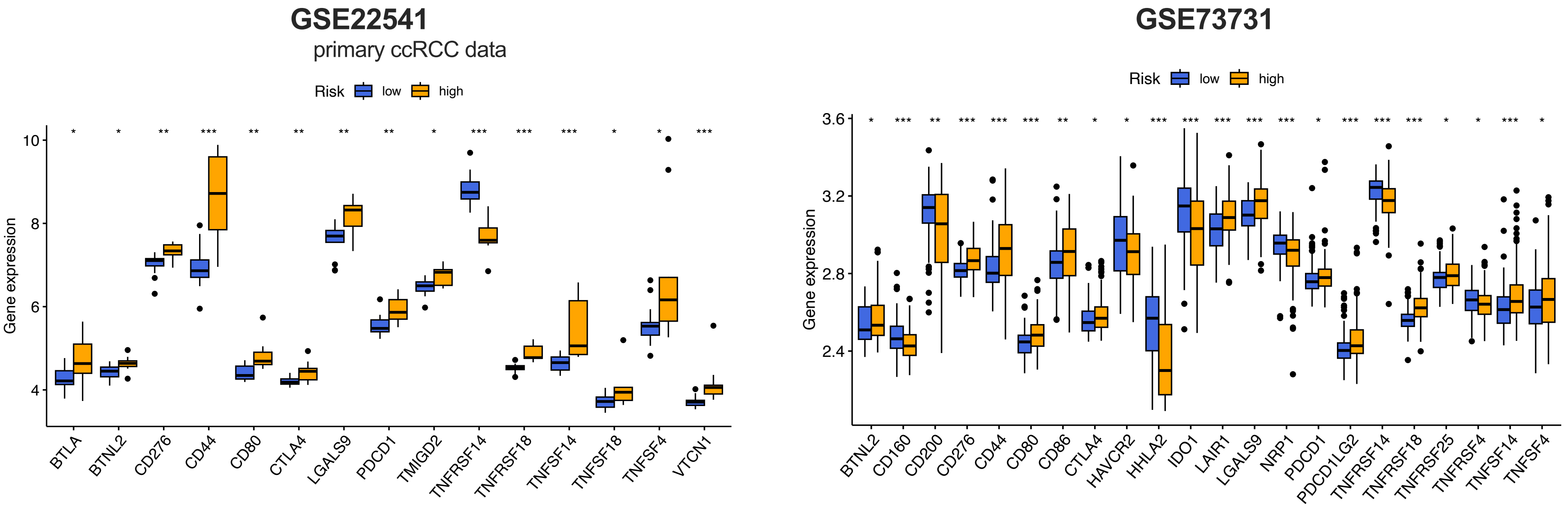
GSE22541
primary ccRCC data
Risk
low
high
Gene expression
BTLA
BTNL2
CD276
CD44
CD80
CTLA4
LGALS9
PDCD1
TMIGD2
TNFRSF14
TNFRSF18
TNFSF14
TNFSF18
TNFSF4
VTCN1
GSE73731
Risk
low
high
Gene expression
BTNL2
CD160
CD200
CD276
CD44
CD80
CD86
CTLA4
HAVCR2
HHLA2
IDO1
LAIR1
LGALS9
NRP1
PDCD1
PDCD1LG2
TNFRSF14
TNFRSF18
TNFRSF25
TNFRSF4
TNFSF14
TNFSF4

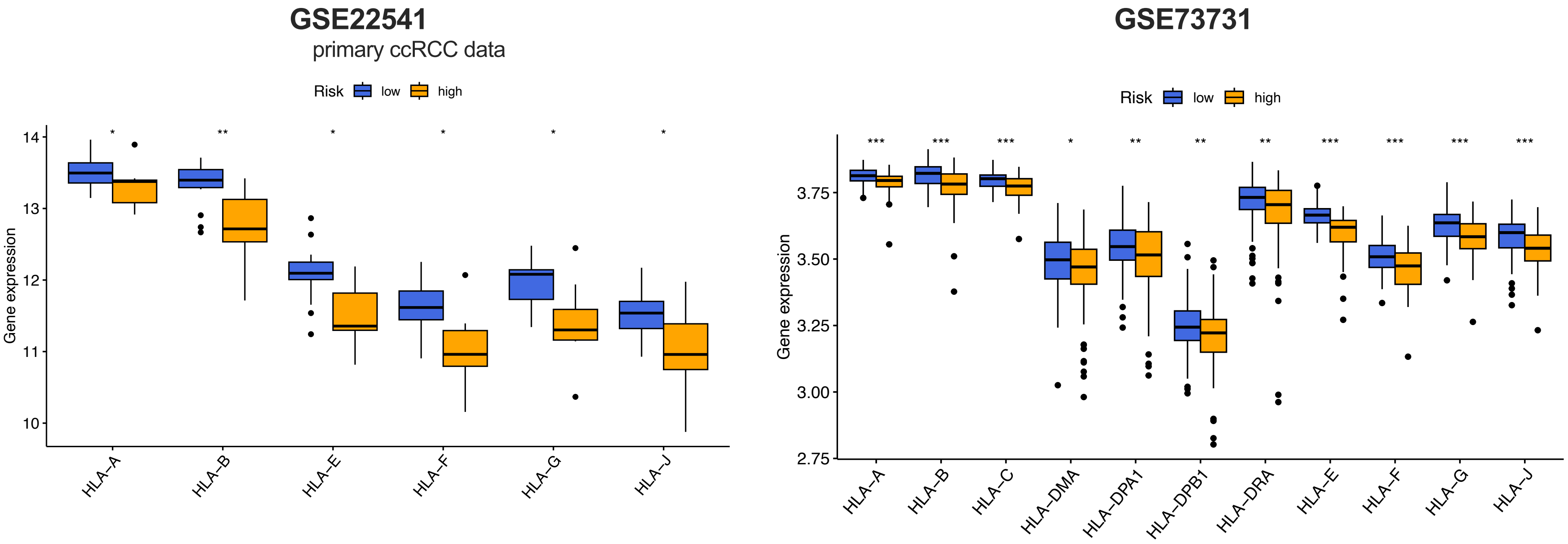
GSE22541
primary ccRCC data
Risk low high
Gene expression
HLA–A
HLA–B
HLA–E
HLA–F
HLA–G
HLA–J
GSE73731
Risk low high
Gene expression
HLA–A
HLA–B
HLA–C
HLA–DMA
HLA–DPA1
HLA–DPB1
HLA–DRA
HLA–E
HLA–F
HLA–G
HLA–J

	pvalue	Hazard ratio
ACC	<0.001	5.222(2.261−12.063)
BLCA	0.141	1.196(0.942−1.517)
BRCA	0.163	0.852(0.680−1.067)
CESC	0.135	0.790(0.580−1.076)
CHOL	0.992	1.004(0.506−1.990)
COAD	0.976	0.992(0.606−1.624)
DLBC	0.583	0.738(0.250−2.181)
ESCA	0.105	0.748(0.526−1.063)
GBM	0.082	1.402(0.957−2.052)
HNSC	0.017	0.818(0.693−0.965)
KICH	0.035	4.633(1.116−19.225)
KIRC	<0.001	2.701(2.040−3.575)
KIRP	0.312	1.385(0.737−2.603)
LAML	0.706	0.937(0.668−1.313)
LGG	0.927	0.975(0.572−1.663)
LIHC	0.012	1.374(1.071−1.763)
LUAD	0.320	1.112(0.902−1.370)
LUSC	0.053	0.829(0.686−1.003)
MESO	<0.001	2.453(1.575−3.820)
OV	0.129	1.190(0.950−1.490)
PAAD	0.405	1.199(0.782−1.837)
PCPG	0.663	0.690(0.130−3.670)
PRAD	0.389	0.550(0.141−2.144)
READ	0.462	1.432(0.551−3.725)
SARC	0.239	1.259(0.858−1.850)
STAD	0.018	0.663(0.472−0.931)
TGCT	0.398	4.282(0.147−125.025)
THCA	0.072	2.187(0.933−5.128)
THYM	0.170	1.969(0.748−5.185)

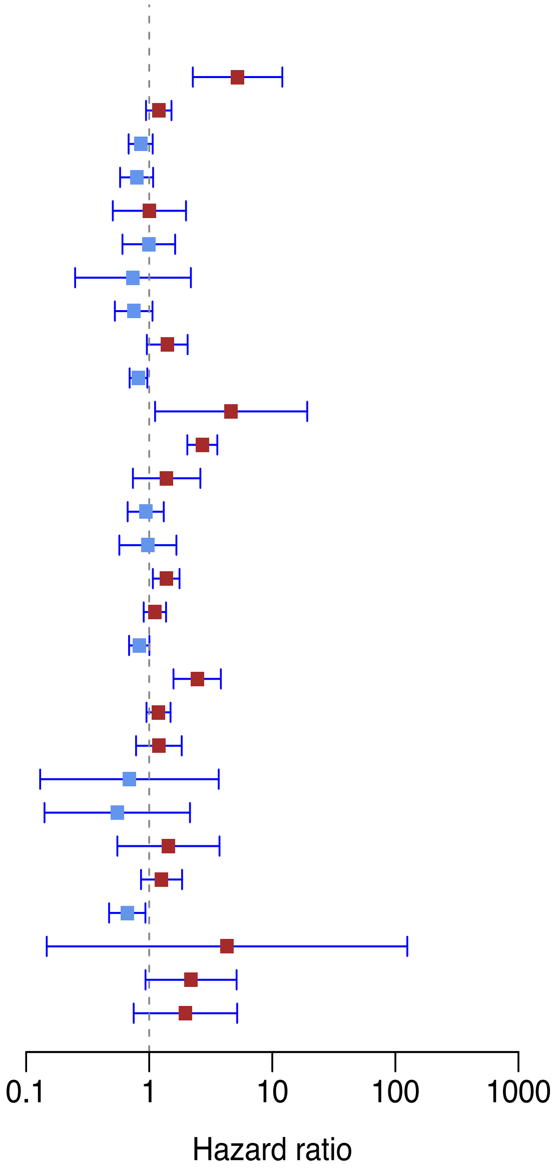

Supplement: Supplementary file 1 — Supplementary figures. [file jcav14p3335s1.pdf]
